# DeepSAGE Reveals Genetic Variants Associated with Alternative Polyadenylation and Expression of Coding and Non-coding Transcripts

**DOI:** 10.1371/journal.pgen.1003594

**Published:** 2013-06-20

**Authors:** Daria V. Zhernakova, Eleonora de Klerk, Harm-Jan Westra, Anastasios Mastrokolias, Shoaib Amini, Yavuz Ariyurek, Rick Jansen, Brenda W. Penninx, Jouke J. Hottenga, Gonneke Willemsen, Eco J. de Geus, Dorret I. Boomsma, Jan H. Veldink, Leonard H. van den Berg, Cisca Wijmenga, Johan T. den Dunnen, Gert-Jan B. van Ommen, Peter A. C. 't Hoen, Lude Franke

**Affiliations:** 1 University of Groningen, University Medical Center Groningen, Department of Genetics, Groningen, The Netherlands; 2 Center for Human and Clinical Genetics, Leiden University Medical Center, Leiden, The Netherlands; 3 Leiden Genome Technology Center, Leiden, The Netherlands; 4 Department of Psychiatry, The Netherlands Study of Depression and Anxiety, VU University Medical Center, Amsterdam, The Netherlands; 5 Department of Biological Psychology, Netherlands Twin Registry, VU University, Amsterdam, The Netherlands; 6 Department of Neurology, Rudolf Magnus Institute of Neuroscience, University Medical Centre Utrecht, Utrecht, The Netherlands; University of Pennsylvania, United States of America

## Abstract

Many disease-associated variants affect gene expression levels (expression quantitative trait loci, eQTLs) and expression profiling using next generation sequencing (NGS) technology is a powerful way to detect these eQTLs. We analyzed 94 total blood samples from healthy volunteers with DeepSAGE to gain specific insight into how genetic variants affect the expression of genes and lengths of 3′-untranslated regions (3′-UTRs). We detected previously unknown *cis*-eQTL effects for GWAS hits in disease- and physiology-associated traits. Apart from *cis*-eQTLs that are typically easily identifiable using microarrays or RNA-sequencing, DeepSAGE also revealed many *cis*-eQTLs for antisense and other non-coding transcripts, often in genomic regions containing retrotransposon-derived elements. We also identified and confirmed SNPs that affect the usage of alternative polyadenylation sites, thereby potentially influencing the stability of messenger RNAs (mRNA). We then combined the power of RNA-sequencing with DeepSAGE by performing a meta-analysis of three datasets, leading to the identification of many more *cis*-eQTLs. Our results indicate that DeepSAGE data is useful for eQTL mapping of known and unknown transcripts, and for identifying SNPs that affect alternative polyadenylation. Because of the inherent differences between DeepSAGE and RNA-sequencing, our complementary, integrative approach leads to greater insight into the molecular consequences of many disease-associated variants.

## Author Summary

Many genetic variants that are associated with diseases also affect gene expression levels. We used a next generation sequencing approach targeting 3′ transcript ends (DeepSAGE) to gain specific insight into how genetic variants affect the expression of genes and the usage and length of 3′-untranslated regions. We detected many associations for antisense and other non-coding transcripts, often in genomic regions containing retrotransposon-derived elements. Some of these variants are also associated with disease. We also identified and confirmed variants that affect the usage of alternative polyadenylation sites, thereby potentially influencing the stability of mRNAs. We conclude that DeepSAGE is useful for detecting eQTL effects on both known and unknown transcripts, and for identifying variants that affect alternative polyadenylation.

## Introduction

Genome-wide association studies (GWAS) have associated genetic variants, such as single nucleotide polymorphisms (SNPs) and copy number variants (CNVs), with numerous diseases and complex traits. However, the mechanisms through which genetic variants affect disease phenotypes or physical traits often remain unclear. To gain insight into these mechanisms, we have combined genotype data with gene expression data by conducting expression quantitative trait locus (eQTL) mapping. Previously, the level of gene expression was primarily assessed using oligonucleotide microarrays, which was a powerful method to profile the transcriptome [1]–[6]. But recently, high-throughput next generation sequencing (NGS) has become available, which allows quantification of expression levels by counting mRNA fragments (RNA-seq) or sequence tags (including serial analysis of gene expression (SAGE), cap analysis of gene expression (CAGE), and massively parallel signature sequencing (MPSS)) [7].

**Figure 1:**
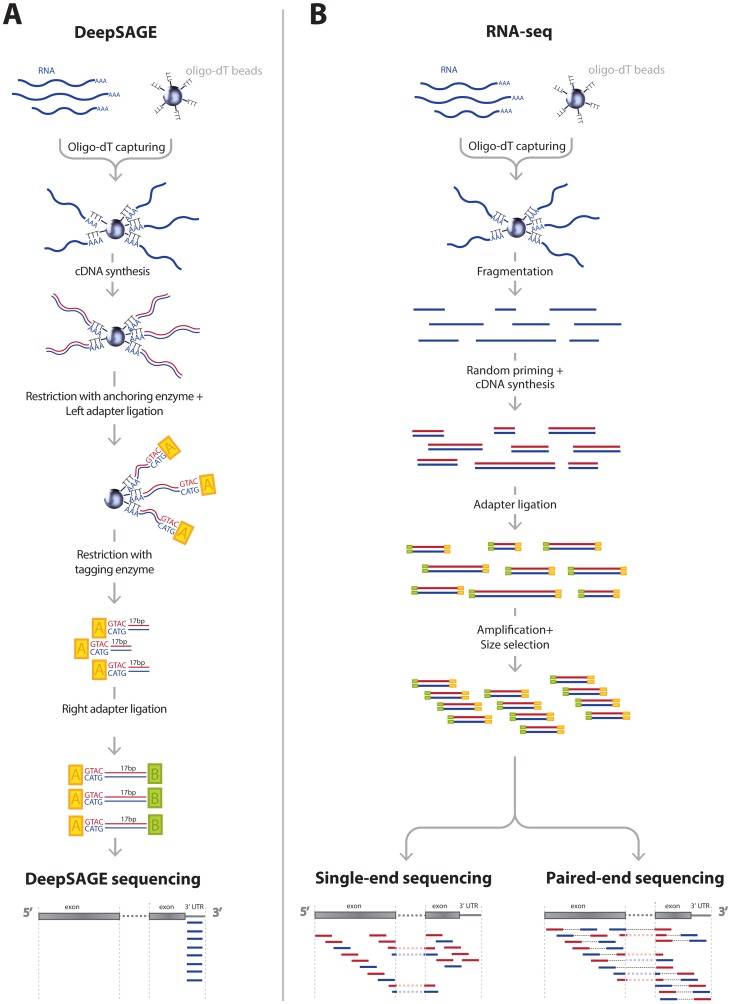
Comparison of typical DeepSAGE and RNA-seq data generation steps. A) DeepSAGE data preparation consists of the following basic steps: after RNA extraction the polyadenylated mRNA fraction is captured with oligo-dT beads. While RNA is still bound to the beads, double-stranded cDNA synthesis is performed. Next, cDNA is digested by NlaIII restriction enzyme (an anchoring enzyme), which cuts the DNA at CATG recognition sequences, leaving only the fragment with the most distal (3′) CATG site associated with the beads. Subsequently, a GEX adapter is attached to the 5′ end. This adapter contains a recognition sequence for the MmeI restriction enzyme that cuts the sequence 17 bp downstream of CATG site. After ligation of a second GEX adapter, fragments containing 21 bp tags (17 unknown nucleotides + CATG) are ready for sequencing. B) A typical protocol for RNA-seq data preparation has the following steps: after RNA extraction the polyadenylated mRNA fraction is captured with oligo-dT beads. Captured RNA is fragmented and for each fragment cDNA synthesis is performed using random hexamer primers. Sequencing adapters are then ligated to each fragment. This is followed by size selection of the DNA fragments and PCR amplification. Then one end of the fragment is sequenced (single-end sequencing) or both ends (paired-end sequencing).

To date, two NGS eQTL studies have been published [8], [9], both of which used RNA-seq. Although RNA-seq is a versatile technique, the coverage in the ultimate 3′-end is usually lower due to the fragmentation and random hexamer priming steps involved in the sample preparation [10] (Figure 1B). DeepSAGE technology [11], [12], however, concentrates on capturing information on the 3′ end of transcripts. In DeepSAGE, enzymatic cDNA digestions generate one specific tag of 17 nucleotides at the most 3′-CATG sequence of each transcript (Figure 1A). The majority of these 21-mer tags (‘CATG’ + 17 nucleotides) can be uniquely mapped to the genome to identify the genes expressed.

There are several features of NGS-based expression quantification methods that are especially important for eQTL mapping. While oligonucleotide arrays target a predefined set of transcripts or exons, both RNA-seq and DeepSAGE are capable of detecting novel and unannotated transcripts. If such a novel gene later turns out to be *cis-*regulated by trait- or disease-associated SNPs, it can represent an interesting causal candidate gene for the trait or disease under investigation. RNA-seq is extremely versatile, as it can quantify the expression of alternative transcripts, which makes it possible to detect SNPs regulating the choice between alternative transcripts. DeepSAGE, however, is generally not suited to detecting alternative splicing because of the 3′ bias of the tag locations [13]. Because only sequence data is generated for these short tags, the read depth per tag is generally much greater than with RNA-seq, permitting accurate quantification of these tags [11], [14]. Thus, this 3′ emphasis makes DeepSAGE suitable for transcript variants that differ in 3′-UTRs and also for detecting alternative polyadenylation events, a widespread phenomenon that generates variation in 3′-UTR length [15], [16]. Shortening or lengthening of the 3′-UTR may result in the loss or gain of regulatory elements, such as miRNA binding sites or binding sites for proteins that can stabilize or destabilize the transcript [17], [18]. Several SNPs that influence the choice for alternative polyadenylation sites have been detected by RNA-seq on a small number of individuals [19]. Here, we analyzed this phenomenon in more depth by performing *cis-*eQTL mapping on DeepSAGE data from total blood samples of 94 individuals.

## Results

### DeepSAGE dataset

For *cis-*eQTL mapping, we used DeepSAGE sequencing of 21 bp tags (16±7 million tags) from total blood samples from 94 healthy, unrelated individuals from the Netherlands Twin Register (NTR) and the Netherlands Study of Depression and Anxiety (NESDA) [20]. Sequence reads were mapped to the reference genome hg19 using Bowtie [21] and assigned to transcripts. We mapped 85±5% of tags to the genome and found that 77±9% of these mapped to exonic regions. Although 66±18% of these reads mapped to hemoglobin-alpha or –beta (*HBA1*, *HBA2*, *HBB*) genes, we were left with sufficient sequencing depth to detect a total of 9,562 genes at a threshold of at least two tags per million.

### *Cis-*eQTL mapping

Once reads had been mapped, we quantified the expression levels of sequenced tags and performed *cis-*eQTL mapping, evaluating only those combinations of SNPs and tags that were located within a genomic distance of 250 kb, while using a Spearman rank correlation test (tag-level false discovery rate (FDR) controlled at 0.05). We identified 540 unique *cis-*regulated tags.

To subsequently increase the statistical power of eQTL detection, we used principal component analysis (PCA) to correct for technical and known and unknown biological confounders. The first principal components (PC) generally capture a high percentage of the expression variation, and these PCs mostly reflect technical, physiological and environmental variability. Removing this variation allows for the detection of more eQTLs [6], [22], [23]. In our data the first principal component significantly correlated with sample GC content, and principal components 7 and 11 correlated with various blood cell count parameters (for details see Text S1, Figures S1 and S2). When using the PC corrected data, we observed an almost two-fold increase in the number of significant *cis-*eQTLs (1,011 unique *cis-*regulated tags, corresponding to 896 unique *cis-*regulated genes at tag-level FDR<0.05). The list of detected eQTLs is given in Table S1.

### Comparison with microarray results

We then compared the DeepSAGE *cis-*eQTLs with *cis*-eQTLs that we had identified using the Affymetrix HG-U219 expression microarrays on the same 94 samples. In that analysis we detected *cis*-eQTLs for only 274 genes (FDR<0.05), only a third of what we identified using DeepSAGE.

**Figure 2:**
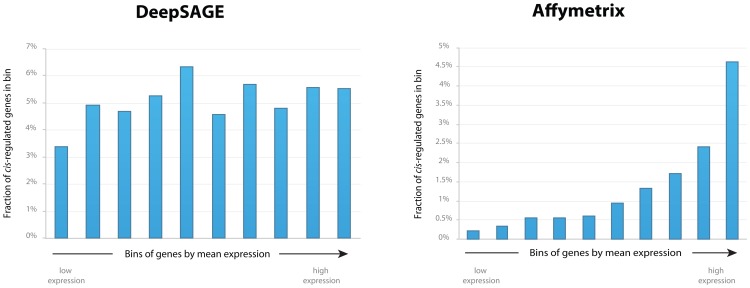
Fraction of *cis-*regulated genes in bins by mean gene expression levels for DeepSAGE and Affymetrix data. For each dataset, all genes were sorted by their mean gene expression levels, and divided into ten equal bins. The X-axis reflects these bins, which are sorted by increasing mean gene expression levels. The Y-axis reflects the fraction of *cis*-regulated genes that fall into each bin.

We observed that this substantial difference could mostly be explained by the fact that the *cis*-eQTLs detected using Affymetrix microarrays nearly always reflected genes that are highly expressed in blood, whereas for DeepSAGE the detected *cis*-eQTL genes had expression levels that could be much lower (Figure 2). Although we only concentrated on tags that were expressed, there was no clear relationship between the mean tag level expression and the probability of showing a significant cis-eQTL. As such, DeepSAGE is much more capable of identifying *cis*-eQTLs for genes showing low expression than conventional microarrays.

It was therefore not a surprise that only 39% of the identified DeepSAGE *cis-*eQTLs could also be significantly detected in the microarray-based dataset (with identical allelic direction) (Figure S3). Indeed, the *cis*-eQTLs that were not replicated in the microarray-based dataset generally had a much lower expression than the replicating *cis*-eQTLs (Wilcoxon Mann Whitney P<2×10^−3^).

And *vice versa*, we could significantly replicate 75% of the detected Affymetrix *cis*-eQTLs with the same allelic direction in the DeepSAGE data (Figure S3), indicating that DeepSAGE shows overlapping results with array-based data. At the same time, this provides insight into the regulation of gene expression by SNPs at many more loci.

We estimated the reduction that could be made in the sample size of the sequencing-based dataset to get the same number of *cis*-regulated genes as in microarray-based data. We observed that the DeepSAGE sample size could be reduced by almost half (to 55 samples) to get the same number of significant *cis*-regulated genes as identified in the microarray analysis of the 94 samples. As such, these results clearly indicate that DeepSAGE has higher statistical power than microarrays.

### *Cis-*eQTL effects on non-coding genes

**Figure 3:**
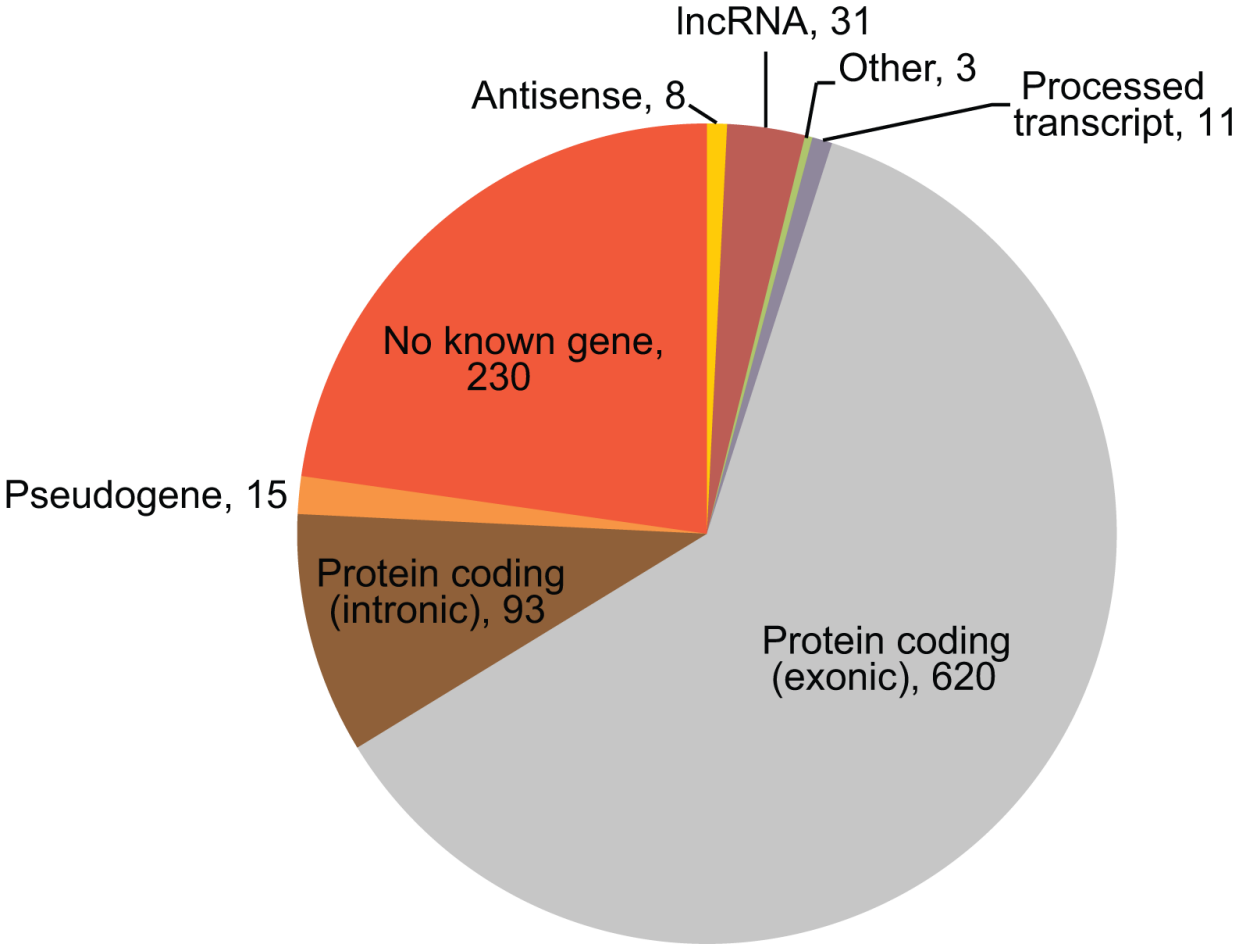
Mapping regions of *cis-*regulated tags. The gene biotypes and exon/intron locations of unique *cis*-regulated tags, according to Ensembl v.69 annotation, are shown. The numbers indicate the number of tags mapping in the genes of the corresponding type.

While most microarray platforms interrogate mainly the protein-coding part of the transcriptome, NGS-based expression profiling will detect the majority of all expressed transcripts. Indeed, we detected eQTLs for known, but non-protein coding, genes: 8 antisense genes and 31 lincRNAs (Figure 3).

We also expected to find a number of *cis*-eQTL effects on previously unknown transcripts. Of the 1,011 tags with a significant *cis*-eQTL effect, 230 did not map to known transcripts. Many of these tags map to retrotransposon-derived elements in the genome, which are known to be a source of novel exons [24]: 73 DeepSAGE tags with significant *cis-*eQTLs that did not map to annotated genes mapped to 72 unique LINE, SINE and LTR elements in the genome (Table 1).

**Table 1:** Number of *cis-*regulated tags mapping to different genomic regions in tag-wise DeepSAGE eQTL mapping.

Type of genomic region	Number of *cis-*regulated tags
**LINE**	32
**SINE**	14
**LTR**	17

### New regulatory roles for disease- and trait-associated SNPs

We checked how many of our *cis-*acting SNPs were associated with complex traits or complex diseases (‘trait-associated SNPs’), as published in the Catalog of Published Genome-Wide Association Studies. 104 of the 6,446 unique trait-associated SNPs were significant *cis*-eQTLs in our data (Table S2).

We were interested to determine whether the DeepSAGE data had revealed *cis*-eQTL effects for trait-associated SNPs that had been missed when using conventional arrays on much larger cohorts. We therefore compared our results to a re-analysis of a large-scale, array-based *cis*-eQTL mapping that we had conducted in whole peripheral blood samples when using a much larger sample size of 1,469 (using Illumina oligonucleotide arrays [6]).

We identified 13 trait-associated SNPs that did show a significant *cis*-eQTL effect in DeepSAGE eQTL mapping, but which did not show a *cis*-eQTL effect in the large, array-based, blood dataset (Table 2). This indicates that many trait-associated SNPs have regulatory effects that will, so far, likely have been missed using microarrays. While some of the tags map in the exons of annotated transcripts, we also found three *cis*-regulated tags in introns (sense direction), two tags antisense to the known transcripts, and two tags outside the annotated transcripts. These results indicate that several trait-associated SNPs affect the expression of previously unknown transcripts, adding functional relevance to SNPs and transcripts that are so far without annotation.

**Table 2:** Trait-associated SNPs affecting DeepSAGE tags of 94 peripheral blood samples, but not detected in an array-based eQTL dataset of 1,469 peripheral blood samples.

SNP name	Tag chr.	Tag position (midpoint)	In gene	Location in gene	Sense/antisense	Closest gene	Repeat masker annotation	Associated trait
rs6704644	2	234380527	*DGKD*	3′-UTR	sense	-	None	Bilirubin levels
rs9875589	3	14196086	*XPC*	intron	antisense	-	LINE L1MB3	Ovarian reserve
rs4580814	5	1050754	*SLC12A7*	3′-UTR	sense	-	None	Hematological and biochemical traits
rs3194051	5	35884591	*None*	-	-	*IL7R*	LINE L2C	Ulcerative colitis
rs4917014	7	50472441	*IKZF1*	3′-UTR	sense	-	None	Systemic lupus erythematosus
rs10092658	8	131017411	*FAM49B*	intron	sense	-	None	Protein quantitative trait loci
rs216345	9	33917317	*UBE2R2*	3′-UTR	sense	-	None	Bipolar disorder
rs12219125	10	20519590	*PLXDC2*	intron	sense	-	SINE AluJb	Diabetic retinopathy
rs7181230	15	40325714	*EIF2AK4*	intron	antisense	-	None	Dehydroepiandrosterone sulphate levels
rs4924410	15	40328035	*SRP14*	3′-UTR	sense	-	None	Ewing sarcoma
rs12594515	15	45995320	*None*	-	-	lincRNA RP11-718O11.1	LTR MLT1A	Waist circumference, weight
rs6504218	17	62400467	*PECAM1*	3′-UTR	antisense	-	None	Coronary heart disease
	17	62397000	*PECAM1*	3′-UTR	antisense	-	LINE L1ME4A	Coronary heart disease
rs4072910	19	8640274	*MYO1F*	intron	sense	-	LINE MER1B	Height

Some newly discovered eQTLs provide novel insights into genome-wide association hits for diseases or physiological traits, e.g. SNP rs216345, which has been associated with bipolar disorder. While it is located just downstream of *PRSS3*, we now saw that it also affects the expression of *UBE2R2*. There are many links between the ubiquitin system and bipolar disorder reported in the literature (e.g. [25], [26]), making *UBE2R2* a more plausible candidate gene for bipolar disorder than *PRSS3*.

### Genes with multiple SAGE tags and opposite allelic direction

**Figure 4:**
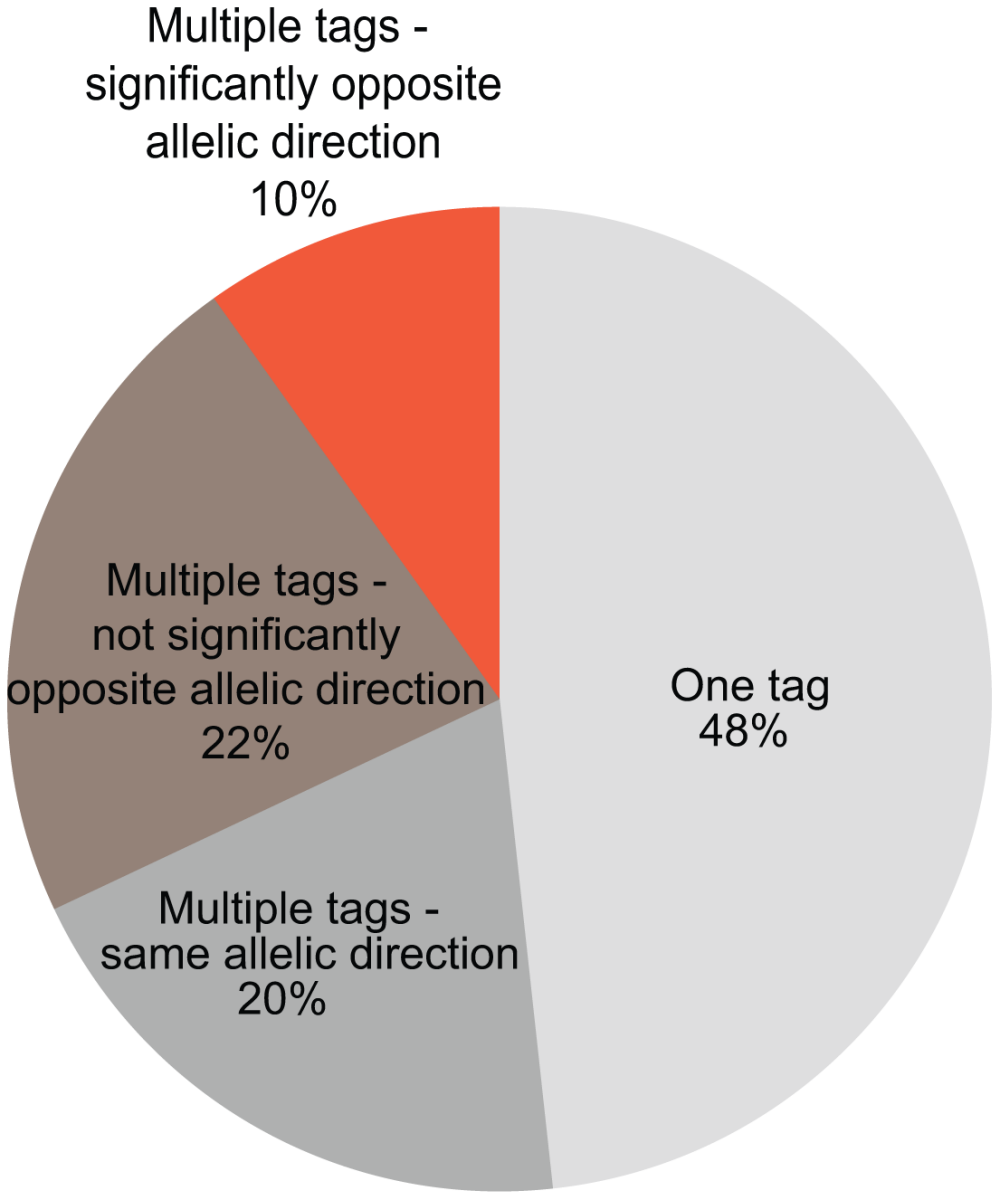
The number of *cis-*regulated tags per gene. The percentages of cis-regulated tags mapping into the same gene are indicated (781 genes overall). For nearly half of the genes (48%) only one tag shows an eQTL effect. If multiple tags map within the same gene, only one eQTL tag should pass the FDR<0.05 significance threshold while the other tag could be less significant. For these eQTLs the allelic direction is shown: same allelic direction (multiple tags within the same gene are cis-regulated by a SNP in the same direction), significantly opposite allelic direction (multiple tags within the same gene are cis-regulated by a SNP but with opposite directions and the difference between the correlation coefficients is significant), or opposite allelic direction but not significant (if the difference between correlation coefficients is not significant).

In DeepSAGE, 21-bp-long cDNA fragments begin at the ‘CATG’ closest to the polyadenylation site (Figure 1). These individual ‘tags’ represent transcripts sharing the same polyadenylation site. If a SNP increases the abundance of one tag of a gene and decreases the abundance of another tag of the same gene, this indicates that the SNP is acting like a switch between transcripts with different 3′-UTRs or between alternative polyadenylation sites [19] (Figure 4).

**Figure 5:**
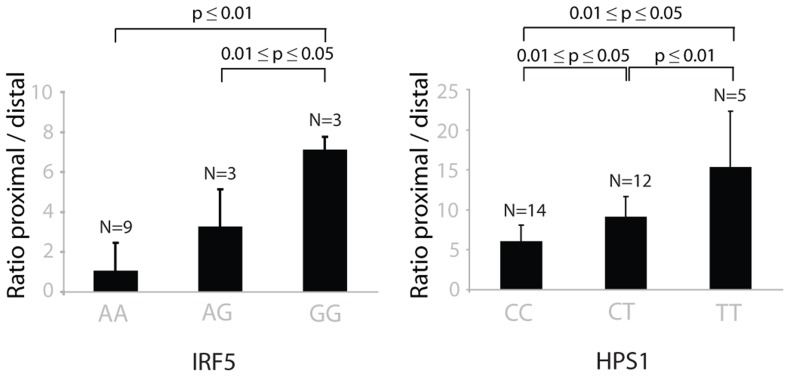
The choice of proximal/distal polyadenylation site in genes *IRF5* and *HPS1* depends on the genotypes of rs10488630 and rs11189600, respectively. The ratio between the abundance of transcripts with proximal and distal 3′-UTR RT-qPCR products in *IRF5* (left) and *HPS1* (right) depends on the genotypes of *cis-*regulating SNPs rs10488630 and rs11189600, respectively. N denotes the number of individuals included in the analysis. These results indicate allele-specific preference for use of a proximal and distal polyadenlyation site.

Twelve genes with highly significant *cis*-eQTLs (p-value<10^−7^) contained tags that were regulated in opposite directions (Table 3). Most of the tags regulated in opposite direction could be explained by switches in alternative polyadenylation sites, as the tags were observed in the same last exon. The effect on alternative polyadenylation in *IRF5* has been found before [19], [27] and was also validated in our cohort by RT-qPCR with primers in the proximal and distal parts of the 3′-UTR (Figure 5). As a further confirmation of the observed switches in using polyadenylation sites, we tested genotype-dependent alternative polyadenylation in two other RNA-seq datasets [8], [9]. In these datasets, we confirmed the effect of two *cis*-regulating SNPs on *THEM4* and *F11R*. However, we could not confirm the effect of other SNPs on targets validated experimentally, including *IRF5*. This shows the limitation of RNA-seq data in detecting alternative polyadenylation events, likely due to limited and unequal coverage of the 3′-UTR. For only two genes, *OAS1* (also reported earlier [28]) and *RP11-493L12.2*, the observed opposite allelic effect originated from transcripts with different last exons, likely due to alternative splicing.

**Table 3:** *Cis-*regulating SNPs significantly* affecting multiple tags of the same gene in opposite directions.

SNP Name	SNP Type	Allele Assessed	Probe Chr.	Probe Center	Overall Z-Score	HGNC Name	Annotation
rs12568757	G/A	G	1	150782318	4.404	*ARNT*	Alternative polyadenylation
			1	150782604	−4.314		
rs12566232	A/C	C	1	151846229	−6.859	*THEM4*	Alternative polyadenylation
			1	151846628	4.292		
rs1062826	G/C	C	1	160965239	−4.46	*F11R*	Alternative polyadenylation
			1	160966976	8.012		
rs13160562	G/A	A	5	96110323	−7.883	*ERAP1*	Alternative polyadenylation
			5	96111908	5.555		
rs3185733	A/C	A	5	112320282	4.027	*DCP2*	Alternative polyadenylation
			5	112356357	−4.46		
rs6948928	T/C	T	7	128589824	8.451	*IRF5*	Alternative polyadenylation
			7	128589265	−7.31		
rs2111903	G/C	C	12	47603121	5.143	*RP11-493L12.2*	Different exon
			12	47599911	−4.676		
rs841718	A/G	G	12	57489368	5.506	*STAT6*	Alternative polyadenylation
			12	57489809	−7.002		
rs2285934	G/T	T	12	113357275	4.931	*OAS1*	Different exon
			12	113355465	−5.191		
rs168822	C/T	T	16	55616984	7.37	*LPCAT2*	Alternative polyadenylation
			16	55620233	−4.664		
rs922446	T/C	T	16	56395733	−4.966	*AMFR*	Alternative polyadenylation
			16	56396100	4.392		
rs1674159	C/T	T	19	5915589	−7.103	*CAPS*	Alternative polyadenylation
			19	5916143	6.126		

* Only significant eQTLs with FDR<0.05 for both *cis-*regulated tags were used.

As we has identified several SNPs that affect alternative polyadenylation, we subsequently used a more permissive strategy, which required that, for a given SNP, only one eQTL tag should pass the FDR<0.05 significance threshold while the other tag could be less significant. However, for such SNP-tag pairs, we then tested whether the allelic directions were opposite and if the difference between correlation coefficients was significant. With a differential correlation significance p-value threshold of 10^−7^, we detected 41 unique genes showing regulation in opposite directions (Table S3). Of these, 23 (56%) showed opposite regulation of two tags in the same annotated 3′-UTR and a further 7 genes (17%) showed opposite regulation of tags in the same exons, both indicative of a switch in polyadenylation sites. Of these we picked *HPS1*, and validated a genotype-determined switch in preferred polyadenylation site usage by RT-qPCR analysis (Figure 5), indicating that the more permissive list also holds genuine changes in polyadenylation sites. The remaining 11 genes showed significant genotype-determined switches in expression of alternative transcripts not sharing the final exon. Thus, switches between shorter and longer 3′-UTRs occur more frequently than switches between transcripts with different 3′-UTRs.

To check whether such results appeared by chance, we took an equal number of top hits from a permuted eQTL run (shuffling the phenotype labels of the expression data, thus breaking the relationship between genotype and expression, but retaining linkage disequilibrium (LD) structure and structure in the expression data) and performed the same analysis as above (assessing an equal number of top eQTLs from the permuted analysis as we had investigated in the real analysis). Using the differential correlation significance threshold of 10^−7^ and conducting this permutation analysis ten times, we did not find any SNP that affected two tags in the same gene in a significantly different way, indicating this method is robust.

**Figure 6:**
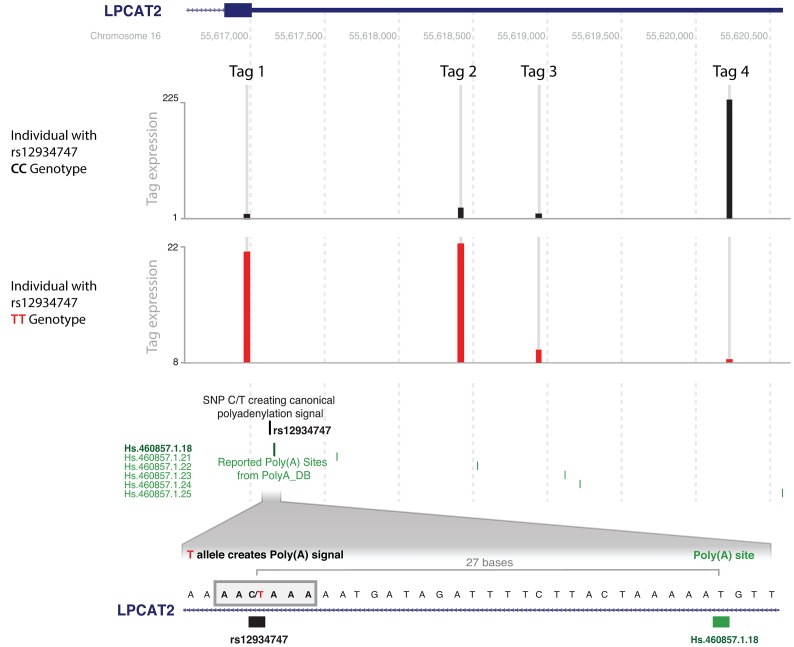
rs12934747*T creates a poly(A) signal in *LPCAT2* and leads to alternative polyadenylation site usage. The y-axis represents the number of counts for the deepSAGE tags. Two samples with different genotypes for SNP rs12934747, CC (reference allele) and TT (alternative allele), are shown as different traces. Below the coverage tracks, the position of rs12934747 is shown, together with the position of all reported polyadenylation sites from polyA_DB. An enlargement of the region containing the SNP is shown below. rs12934747 is located at the beginning of the 3′-untranslated region (3′-UTR) of *LPCAT2*, 27 nucleotides upstream a reported and experimentally validated polyadenylation site. This SNP changes the sequence, creating a polyadenylation signal that leads to the usage of the reported polyadenylation site. The square block indicates the sequence of the polyadenylation signal created by rs12934747. The creation of this signal shortens the 3′-UTR, as indicated by the higher abundance of the proximal DeepSAGE just upstream of the polyadenylation signal, and the nearly absent distal DeepSAGE, in the sample with the TT genotype (both tags indicated by arrows). Tag 2 was filtered out because it was expressed in less than 90% of individuals. There is an additional tag 3 in-between the proximal and distal tags, which is not *cis*-regulated.

Since the eQTL SNPs are usually in strong LD with multiple SNPs, it is difficult to conclude whether a SNP is causal or which SNP is the likely causal variant. To identify the likely causal variant, we assessed whether any of these SNPs caused changes in polyadenylation site usage. A direct effect on alternative polyadenylation can be explained by a change in the polyadenylation site (corresponding to the cleavage site) or in the polyadenylation signal (a six-nucleotide motif located between 10–30 bases upstream of the cleavage site). We searched for likely causative SNPs in linkage disequilibrium with the polyA-QTL SNP (R^2^≥0.8). We did not find any strong evidence for SNPs influencing the cleavage site and focused on *cis*-regulating SNPs located within polyadenylation signals. Considering the length and the motif of canonical and non-canonical polyadenylation signals [15], we performed a motif analysis in the sequence surrounding each *cis*-regulating SNP. We identified five SNPs that likely affect polyadenylation because there was a change in the polyadenylation signal (Table 4). As previously shown, rs10954213 causes the formation of a stronger polyadenylation signal in *IRF5*. Similar changes from non-canonical to stronger, canonical polyadenylation signals were observed for rs1062827 in *F11R* and rs6598 in *GIMAP5*. Moreover, rs12934747 creates a new canonical AATAAA polyadenylation signal in *LPCAT2*. The presence of this alternative polyadenylation signal at the beginning of the 3′-UTR leads to a decrease in transcripts containing the full length 3′-UTR, as observed by DeepSAGE (Figure 6). An opposite effect is observed for rs7063 in the ultimate 3′-end of the *ERAP1* gene, where the SNP causes the disruption of the strong canonical motif, and results in the use of a more proximal polyadenylation signal. Unfortunately we were not able to identify likely causative SNPs for each of these eQTLs. This could have several reasons: we imposed strict thresholds (R^2^≥0.8) on the LD between the detected *cis-*eQTLs and the putative causative SNPs; by imputing to the 1000 genomes dataset we may have missed causative SNPs unique to the Dutch population; and the list of experimentally validated polyadenylation sites is not exhaustive, because their detection depends on the expression level and cell type analyzed.

**Table 4:** SNPs that likely affect polyadenylation due to a change in the polyadenylation signal.

Cis-regulating SNP	Causal SNP	R^2^	SNP type	Gene	Reference sequence	Alternative polyA signal	Distance to polyA site (bp)	Effect on 3′-UTR length
***Formation/activation of polyA signal***								
rs6948928	rs10954213	0.76*	G/A	*IRF5*	AATGAA	AATAAA	15	Shortening
rs168822	rs12934747	0.87	C/T	*LPCAT2*	AACAAA	AATAAA	27	Shortening
rs1062826	rs1062827	0.99	G/A	*F11R*	AGTAAA	AATAAA	21	Shortening
rs759011	rs6598	1	G/A	*GIMAP5*	AATAGA	AATAAA	13	Shortening
***Disruption of polyA signal***								
rs13160562	rs7063	1	T/A	*ERAP1*	AATAAA	AAAAAA	23	Shortening

* This SNP was reported in [27] and is validated by our data.

Seven of the SNPs affecting polyadenylation are reported in the GWAS catalog as associated with diseases (Table S3), including rs2188962 and rs12521868, which are associated with Crohn's disease. We found that these SNPs were associated with a switch in the polyadenlyation site of *IRF1*. This may reinforce previous evidence that *IRF1* is the gene in the IBD5 locus responsible for its association with Crohn's disease [29]. *IRF1* is a family member of the *IRF5* gene. Thus, in the family of interferon regulatory factors, we found two members with genetic regulation of alternative polyadenylation sites, likely explaining susceptibility for Crohn's disease and systemic lupus erythematosus, respectively.

Another example is rs3194051, located in the *IL7R* gene. This SNP was not found in the analysis described above since it affects the expression of a tag on the same strand, downstream of *IL7R* in a LINE element (Table 2). However, this tag may represent an alternative 3′-UTR for IL7R. The SNP is associated with ulcerative colitis and IL7R may be another example of a gene in the inflammatory response pathway demonstrating alternative polyadenylation.

### Meta-analysis with RNA-seq data

In order to increase the statistical power to detect associations of SNPs with gene expression, we performed a first-of-its-kind eQTL mapping meta-analysis, combining DeepSAGE data with two published RNA-seq datasets. We used paired-end sequencing of mRNA derived from lymphoblastoid cell lines from HapMap individuals of European origin [8] and 35 and 46 bp single-end sequencing of mRNA derived from lymphoblastoid cell lines from HapMap individuals of Yoruba origin [9]. Sequence reads were mapped to the reference genome hg19 using Tophat [30] and assigned to transcripts. A consistently high percentage of reads (86–87% of aligned reads) mapped to exonic regions (Table 5).

**Table 5:** Description of RNA next generation sequencing datasets.

Dataset		Sequencing type	Cell tissue type	Number of samples	Read length	Million reads per sample	Average % of mapped reads	Average % of mapped reads mapping to exons
**Montgomery ** ***et al.***		Paired-end RNA-seq	LCL	60	37 bp	9.5±3	56	87
**Pickrell ** ***et al.***	**Yale**	Single-end RNA-Seq	LCL	72	35 bp	8.1±2.3	85	86
**Pickrell ** ***et al.***	**Argonne**	Single-end RNA-Seq	LCL	72	46 bp	8.1±1.8	80	86
**NTR-NESDA**		DeepSAGE	Total blood	94	21 bp	16±7	85	88

We first performed eQTL mapping separately in all three datasets (Table 6), summarizing expression on the transcript level to permit comparisons between the datasets. The numbers of *cis*-regulated genes detected in transcript-wise analysis was lower than in tag-wise analysis, possibly because we missed resolution on alternative splicing- and alternative polyadenylation events. Again, PC correction greatly improved the number of *cis*-eQTLs detected (Table 6).

**Table 6:** Number of detected *cis-*eQTLs in transcript-wise analysis of three harmonized RNA NGS datasets.

	Number of unique genes with cis-eQTLs	
	Without principal component correction	With principal component correction
**Montgomery ** ***et al.*** ** (paired** ***-end RNA-seq*** **)**	94	145
**Pickrell ** ***et al.*** ** (** ***single-end RNA-seq*** **)**	199	438
**NTR-NESDA transcript-wise ** ***(DeepSAGE)***	292	579
**Meta-analysis**	651	1,207

We applied PC correction to the individual datasets. As for the DeepSAGE analysis, the first PC correlated strongly with the mean GC-percentage in the two RNA-seq datasets (Figure S1). We then assessed the robustness of the identified *cis*-eQTLs: we checked whether those in one dataset could be significantly replicated in the other two datasets. We observed that in each of the RNA-seq datasets approximately one-third of *cis-*eQTLs could be replicated in the other dataset (Table S4). The overlap between RNA-seq and DeepSAGE was smaller, reflecting differences in the two technologies, in cell types and in populations. In each comparison, we observed a very high concordance in the allelic direction of *cis*-eQTLs that could be replicated in another dataset.

We also looked at the replication of RNA-seq eQTLs in corresponding micro-array-based datasets. 80–88% of such eQTLs could be replicated in microarray data (Table S5).

As we could cross-replicate many *cis*-eQTLs, we decided to conduct a meta-analysis to increase the statistical power. We calculated joint p-values using a weighted Z-score method. The number of *cis-*regulated genes then increased to 1,207 unique genes (Table 6) (a list of detected eQTLs is given in Table S6), indicating that a meta-analysis of different types of sequencing-based eQTL datasets reveals many more *cis*-regulated genes than the individual analyses.

For our meta-analysis results we determined the number of disease- and trait-associated SNPs using the Catalog of Published Genome-Wide Association Studies in the same way as for the DeepSAGE dataset. 107 of the 6,446 unique trait-associated SNPs showed a significant *cis*-eQTL effect in the meta-analysis. The overlap with 104 trait-associated SNPs detected in tag-wise DeepSAGE eQTL mapping was 37, indicating that the DeepSAGE transcript end quantification revealed other trait-associated *cis*-eQTLs than a meta-analysis on the level of whole transcripts. 21 of the 107 SNPs showed a significant *cis*-eQTL effect in the sequencing-based meta-analysis, but did not show a *cis*-eQTL effect in the large array-based blood dataset (Table 7).

**Table 7:** Trait-associated SNPs detected in the sequencing-based transcript-wise meta-analysis, but not detected in array-based eQTL dataset of 1,469 peripheral blood samples.

SNP name	Chr.	Transcript position (midpoint)	*Cis-*regulated gene	Associated trait
rs1052501	3	41963564	*ULK4*	Multiple myeloma
rs347685	3	141782879	*TFDP2*	Chronic kidney disease
rs4580814	5	1081324	*SLC12A7*	Hematological and biochemical traits
rs4947339	6	28911984	*C6orf100*	Platelet aggregation
rs2517532	6	31024818	*HCG22*	Hypothyroidism
rs2844665	6	31024818	*HCG22*	Stevens-Johnson syndrome and toxic epidermal necrolysis (SJS-TEN)
rs6457327	6	31024818	*HCG22*	Follicular lymphoma
rs3130501	6	31324124	*HLA-B*	Stevens-Johnson syndrome and toxic epidermal necrolysis (SJS-TEN)
rs2858870	6	32434437	*HLA-DRB9*	Nodular sclerosis Hodgkin lymphoma
rs3129889	6	32434437	*HLA-DRB9*	Multiple sclerosis
rs3135388	6	32434437	*HLA-DRB9*	Multiple sclerosis
rs477515	6	32434437	*HLA-DRB9*	Inflammatory bowel disease
rs9271100	6	32524134	*HLA-DRB6*	Systemic lupus erythematosus
rs9273349	6	32632106	*HLA-DQB1*	Asthma
rs3807989	7	116183034	*CAV1*	PR interval
rs12680655	8	135604552	*ZFAT*	Height
rs4929923	11	8642408	*TRIM66*	Menarche (age at onset)
rs12785878	11	71161461	*RP11-660L16.2*	Vitamin D insufficiency
rs12580100	12	56436876	*RPS26*	Psoriasis
rs4924410	15	40329664	*SRP14*	Ewing sarcoma
rs7364180	22	42184613	*MEI1*	Alzheimer's disease biomarkers

## Discussion

We have described the results from *cis-*eQTL mapping on DeepSAGE sequencing, a technique that is different from RNA-seq since it mainly targets the 3′-end of transcripts. We identified 1,011 unique *cis-*regulated tags (significant at tag-level FDR<0.05).

We performed eQTL mapping on the microarray expression data of the same samples and the number of detected *cis-*eQTLs was much smaller than in the DeepSAGE data, indicating the higher power of DeepSAGE in eQTL mapping. Moreover, for 220 of the *cis*-eQTLs SNPs detected by DeepSAGE we did not detect a significant *cis*-eQTL in a much larger microarray-based study in 1,469 whole peripheral blood samples [6]. 13 of these SNPs were reported as disease- or trait-associated in the GWAS catalog.

We observed that the number of *cis-*eQTLs detected in microarray data was higher in highly expressed genes, whereas for DeepSAGE the detected *cis*-eQTL genes had expression levels that could be much lower (Figure 2). This means that DeepSAGE is much better at identifying *cis*-eQTLs for genes showing low expression than conventional microarrays. This is because gene expression quantification using microarrays is more difficult as there is always a background signal present that needs to be accounted for. This is not the case for next-generation sequencing: although stochastic variation plays a major role in determining what RNA molecules will eventually be sequenced (especially for transcripts of low abundance), detection of such an RNA molecule is direct proof that it is being expressed.

Clearly, DeepSAGE can capture events that are likely to be missed by RNA-seq and conventional microarrays. It is not surprising, due to the different emphasis of DeepSAGE, that we could only replicate 39% of the DeepSAGE *cis*-eQTLs in the microarray data with a consistent allelic direction (Figure S3). The limited overlap between DeepSAGE- and microarray-based eQTL studies may be partly explained by the fixed thresholds applied, the interrogation of different transcript variants, and by the smaller dynamic range of microarrays. In addition, we found that more highly expressed genes were more often replicated than lower expressed ones.

Moreover, DeepSAGE allows for the detection of non-coding and novel transcripts not present on the microarrays. We showed that genetic variation affects the expression of a substantial number of lincRNAs and antisense genes, some of which have been linked to clinical traits. This suggests that clinical traits may be modified by expression of antisense transcripts or alternative 3′-UTR selection, which are not separately quantified in the microarray-based studies or in most RNA-seq, where standard protocols are still not strand-specific.

We also noticed a relatively high proportion of eQTLs with DeepSAGE tags mapping in SINE, LINE and LTR elements. These transposable elements contribute to the evolution and inter-individual variation of the human genome and to the diversification of the transcriptome, the latter facilitated by their inherent potential to be transcribed and the presence of cryptic splice acceptor and donor sites [24], [31], [32]. Some of the DeepSAGE tags we identified may be located in entirely new transcripts, but the majority is likely to represent alternative exons or 3′-UTRs of known transcripts, in accordance with the observed preferential location in introns or near genes.

Associations with transcripts and transcript variants not yet annotated may help to discover a function for these transcripts, as they are likely to play a role in the physiological and clinical traits associated with the SNP. Moreover, this will complement our knowledge of the pathways associated with these physiological and clinical traits.

In our study, we have demonstrated that genotype-dependent switches in the preference of alternative polyadenylation sites are common. One of these events has been well characterized: SNP rs10954213 creates an alternative polyadenylation site in *IRF5*, shortens the 3′-UTR, stabilizes the mRNA, and increases *IRF5* expression, explaining its genetic association with systemic lupus erythematosus [19], [27]. We have now discovered more examples where SNPs create or disrupt polyadenylation motifs. Amongst others, we identified a new, similar, genotype-dependent switch in preferred polyadenylation site for family member *IRF1*, with a probable link to Crohn's disease. Alternative polyadenylation associated with shortening of 3′-UTRs is a prominent event in the activation of immune cells [18]. Thus, genetically determined use of a proximal polyadenylation sites may predispose to inflammatory disorders such as Crohn's disease. The opposite correlations that we observed for most genes were slightly less pronounced than for *IRF5*. This indicates that mechanisms other than the creation or disruption of canonical polyadenylation motifs may also play a role. For example, SNPs in miRNA or protein-binding sites may specifically affect the stability of the transcript variant with the long 3′-UTR.

We subsequently conducted a *cis-*eQTL meta-analysis on the heterogeneous types of data using methods extended from those we developed for microarray-based eQTL meta-analysis [6]. We identified 1,207 unique *cis-*regulated genes. This number is substantially higher than in each of the datasets separately and indicates that different protocols for digital gene expression generally deliver consistent results. Nevertheless, the overlap at a fixed FDR of 0.05 is rather small, in particular between DeepSAGE and RNA-seq data. While this is partly attributable to using a strong threshold, there are other important reasons: firstly, the RNA-seq and DeepSAGE technologies frequently interrogate different transcript variants. Secondly, the RNA-seq studies were done on lymphoblastoid cell lines (LCLs) while the DeepSAGE study was on total blood, and some *cis-*eQTLs may be tissue-specific [33], [34]. Finally, the DeepSAGE technology is strand-specific but the RNA-seq technologies evaluated here are not: where DeepSAGE will evaluate the expression of sense and antisense transcripts separately, RNA-seq will sum them. These reasons could explain why the percentage of RNA-seq-derived eQTLs that can be replicated by DeepSAGE is higher than the percentage of DeepSAGE-derived eQTLs that can be replicated by RNA-seq.

We conclude that DeepSAGE technology is useful to determine *cis*-eQTLs, as it is able to quantify the expression of novel transcripts, and to detect alternative polyadenylation effects and alternative 3′-UTR selection. It is complementary to other sequencing-based approaches, as they each reveal slightly different regulatory effects of genetic variants. Different sequencing-based eQTL analyses generally deliver consistent results, allowing for meta-analyses across different technologies. Future eQTL mapping studies based on DeepSAGE and other next generation sequencing strategies, using larger cohorts and different techniques, will likely reveal a more comprehensive picture of how far genetic variation affects the expression of protein-coding genes and non-coding RNAs.

## Materials and Methods

### Ethics statement

The medical ethical committee of the VUMC, Amsterdam, the Netherlands, approved the collection and analysis of material blood, DNA and RNA from the 94 participants from the Netherlands Twin Registry (NTR) and the Netherlands Study of Depression and Anxiety (NESDA).

### NTR-NESDA dataset

We analyzed 21 bp DeepSAGE data from total blood RNA of 94 unrelated individuals who participated in NTR or NESDA. RNA was isolated using PaxGene tubes [20], [35], [36]. DeepSAGE sample preparation protocols, and alignment approaches were described in [37]. One sample was run on one lane of the Illumina GAII instrument. Data are available in ArrayExpress under accession number E-MTAB-1181.

The NTR-NESDA data was imputed using Beagle v3.1.0, with HapMap2 release 24 as a reference.

### Tag mapping and expression estimation

Tags from DeepSAGE sequencing were aligned to the NCBI build 37 reference genome using Bowtie v. 0.12.7 allowing for a maximum of 1 mismatch and a maximum of 2 possible alignments (-n 1 -k 1 -m 2 –best –strata options).

The expression values were both quantified on an individual tag and transcript level. For the tag-wise analysis, the total number of occurrences of each unique tag in each sample was counted. We only included tags that were present in >90% of samples. Tags with SNPs in the CATG recognition sequence (according to dbSNPv135) and the transcripts containing those tags were removed before eQTL analysis, since these SNPs can affect the position of the SAGE tag in the transcript. For the transcript-wise analysis, the tag counts for tags overlapping the exons of a transcript by at least half of the tag length were summed.

Coordinates of LINE, SINE, LTR elements were derived from UCSC's RepeatMasker track (update: 2009-04-24).

### GC content bias estimation

To calculate the GC content per individual for DeepSAGE data, GC frequencies for all mapped tags were summed after excluding the twenty most abundant tags, since their high abundance would give biased estimates.

### *Cis*-eQTL mapping and correction for confounding effects through principal component analysis

Before eQTL mapping, transcript and tag expression values were quantile normalized. To perform cis-eQTL mapping, association of SNPs with the expression levels of tags or transcripts within a distance of 250 kb (as this is the average size of linkage regions) of the midpoint of the transcript or tag were tested with a non-parametric Spearman's rank correlation. Multiple testing correction was performed, controlling the false discovery rate (FDR) at 0.05. To determine the FDR, we created a null distribution by repeating the cis-eQTL analysis after permuting the sample labels 10 times [38].

We argue that gene expression levels from NGS-based datasets are, like micro-array based data, derived from genetic, technical and environmental effects. As such, compensating for these non-genetic effects would increase the power to detect *cis*-eQTL effects. To mitigate the effects of non-genetic sources of variability, we first log_2_ transformed the data and centered and scaled each tag, and subsequently applied PCA on the sample correlation matrix. We then used the first PCs as covariates, and re-did the non-parametric cis-eQTL mapping on the residual expression data (using the procedure described by [6]).

### Validation of genotype-dependent alternative polyadenylation in RNA-seq datasets

The genomic coordinates of the 3′-UTR, obtained from Refseq Genes, were split into two separate regions (distal and proximal 3′-UTRs) according to the position of the DeepSAGE tags with opposite directions, the position of LongSAGE tags from CGAP, and the position of reported and predicted polyadenylation sites from polyA_DB database. To calculate the coverage in proximal and distal regions in RNA-seq datasets, we created a coverage histogram from each .bam alignment file using coverageBed tool from BEDTools package (version 2.17.0) [39]. Subsequently, a custom Python script was used to convert the histogram in number of nucleotides mapped per region, normalized by the length of the region. The ratio between the number of counts in the proximal region and the distal region was then calculated.

### qPCR validation of alternative polyadenylation

Expression of short and long variants of *HPS1* and *IRF5* was quantified by qRT-PCR, which was performed on a subset of RNA samples used for the DeepSAGE sequencing. cDNA was synthesized from 400 ng of total RNA using BioScript MMLV Reverse Transcriptase (Bioline) with 40 ng of random hexamer and oligo(dT)18 primers following manufacturer's instructions (for the list of primer sequences see Table S7). Primers specific to short or long variants of *HPS1* were designed using Primer3Plus program, primers for *IRF5* were designed as previously described [40]. qRT-PCR was performed on the LightCycler 480 (Roche) using 2× SensiMix reagent (Bioline). 45 cycles of two-step PCR were performed for *HPS1*, and 55 cycles of three-step PCR (95°C for 15 s, 48°C for 15 s, and 60°C for 40 s) for *IRF5*. Each measurement was performed in duplicates. PCR efficiency was determined using the LinRegPCR program [41] v.11.1 according to the described method [42]. Ratios between distal and proximal PCR products were then calculated and significance was tested performing a T-test.

### Identifying causal SNPs affecting polyadenylation

We obtained all the proxy SNPs for all SNPs identified as *cis-*regulating the choice of polyadenylation site. To do this we used bi-allelic SNPs that pass QC from the 1000G European panel (v3.20101123) and took all SNPs that were in linkage disequilibrium with the query SNPs (R^2^≥0.8, distance between SNPs within 250 kb).

From this list of *cis*-regulating SNPs in linkage disequilibrium, we kept only SNPs, which were located in the *cis*-regulated genes. The filtering was performed by intersecting .bed files containing SNPs coordinates and coordinates of cis-regulated genes from RefSeq database, using table browser tool in UCSC genome browser and the overlap intervals tool in Galaxy (version 1.0.0).

Intersection of SNPs with validated and predicted polyadenylation sites was performed using annotation in the PolyA-DB database (PolyA_DB 1 and PolyA_SVM) on UCSC (table browser tool). Detection of SNPs within polyadenylation signals was performed by extracting the strand specific sequence five nucleotide upstream and downstream each SNP (using table browser tool in UCSC) and performing a motif search using custom Perl script. Canonical and non-canonical polyA motifs searched were AATAAA, ATTAAA, TATAAA, AGTAAA, AAGAAA, AATATA, AATACA, CATAAA, GATAA, AATGAA, TTTAAA, ACTAAA, and AATAGA. For every SNP located in a putative polyadenylation signal motif, the distance to validated and predicted polyadenylation sites from PolyA-DB was calculated. Only motifs within a distance of 30 nucleotides from a polyadenylation site were considered true polyadenylation signals. Newly formed polyadenylation signals were detected by changing the reference allele of the SNP with the alternative allele, followed by the same polyadenylation signal motif search using custom Perl scripts.

For the *cis*-regulated genes where the SNP is located within a true polyadenylation signal, we retrieved the coverage of every SAGE tag upstream and downstream the putative affected polyadenylation site and calculated the ratio between proximal and distal tags for the different genotypes to confirm the expected effects of polyadenylation site formation or disruption.

### RNA-seq datasets

For the meta-analysis we combined DeepSAGE data with two published RNA-seq datasets. The first dataset was 37 bp paired-end RNA-sequencing data from HapMap individuals ([8], [ArrayExpress:E-MTAB-197]): RNA from lymphoblastoid cell lines of 60 HapMap CEPH individuals was sequenced on the Illumina GAII sequencer, while genotype data had already been generated within the HapMap project.

The second dataset was single-end RNA-sequencing data from HapMap individuals [9], [43] [GEO:GSE19480 and at http://eqtl.uchicago.edu/RNA_Seq_data/]: RNA was sequenced from lymphoblastoid cell lines of 72 HapMap Yoruba individuals from Nigeria on the Illumina GAII platform in two sequencing centers: Yale (using 35 bp reads) and Argonne (using 46 bp reads).

Since the Montgomery *et al.* paper used genotype data for some individuals that were not in the HapMap3 panel (NA0851, NA12004, NA12414 and NA12717), we imputed these individuals using Beagle v3.1.0, with HapMap2 release 24 as a reference.

### RNA-seq read mapping

Reads from single- and paired-end RNA-sequencing were mapped to the human genome NCBI build 37 (reference annotation from Ensembl GRCh37.65) using Tophat v. 1.3.3 [30] – a splice-aware aligner that maps RNA-seq reads to the reference genome using Bowtie [21]. We used default settings (maximum 2 mismatches, 20 possible alignments per read) with a segment length value of 17 bp. Reads that corresponded to the flag 1796 in the .bam alignment file (read unmapped, not primary alignment, read fail quality check, read is PCR or optical duplicate) were filtered out. The numbers of raw and mapped reads for each dataset are given in Table 5.

### Read quantification

To estimate expression levels in RNA-seq data, reads that overlapped with exons from known transcripts (GRCh37.65) were quantified using the coverageBed method from BEDTools suite [39].

For transcript level quantification the read count 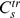 for sample *s* for transcript *tr* was calculated as a sum of expression values over all exons contained in this transcript:
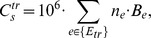
where


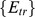
 – set of all exons of transcript *tr*,


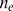
– number of reads overlapping exon *e* by not less than half of read's length,


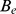
– breadth of coverage for exon *e* (% of exon length covered by the reads mapping to that exon).

In case a read mapped to multiple transcripts, the read was counted for all transcripts, since the short reads are difficult to assign to a specific transcript. Multiplication by breadth of coverage was performed to help in distinguishing between different isoforms by assigning higher weight to exons fully covered by reads in contrast to alternative exons covered only partly.

Because different methods have different capacity to identify alternative splicing events, we subsequently summarized our eQTL results to unique genes.

### Meta-analysis

Meta-analysis was conducted by using a weighted Z-method, weighing each of the datasets by the square root of the number of samples per dataset [6].

### Microarray datasets

We compared the results to corresponding microarray dataset eQTL mapping results. For each of the 94 individuals from NTR-NESDA study, Affymetrix HG-U219 expression data were generated at the Rutgers University Cell and DNA Repository (RUCDR, http://www.rucdr.org). NTR and NESDA samples were randomly assigned to plates with seven plates containing subjects from both studies to better inform array QC and study comparability. Gene expression data were required to pass standard Affymetrix QC metrics (Affymetrix expression console) before further analysis. Probe sets were removed when their mapping location was ambiguous or if their location intersected a polymorphic SNP (dropped if the probe oligonucleotide sequence did not map uniquely to hg19 or if the probe contained a polymorphic SNP based on HapMap3 [44] and 1000 Genomes [45] project data). Expression values were obtained using RMA normalization implemented in Affymetrix Power Tools (APT, v 1.12.0). MixupMapper revealed no sample mix-ups [46].

For RNA-seq data we used corresponding microarray datasets that were available for most of the individuals present in RNA-seq datasets. We used Illumina expression data provided by Stranger *et al*. [3] of the 72 HapMap YRI individuals (56 of which were also present in RNA-seq dataset from Pickrell *et al.*) and 60 HapMap CEU individuals provided by Montgomery *et al.* (58 of which were also present in RNA-seq dataset from Montgomery *et al.*).

The same normalization procedure was performed as for the sequencing-based datasets: quantile normalization, and subsequent probe set centering to zero, z-score transformation, and scaling to a standard deviation of one.

### Data access

The newly generated DeepSAGE data for NTR-NESDA dataset is available in ArrayExpress under accession number E-MTAB-1181 (ENA: ERP001544).

## Supporting Information

Figure S1 Correlation of GC content with principal component 1 (PC1) eigenvector coefficients for all the three datasets. Pearson correlation coefficient and corresponding p-value are shown in the plot.
(EPS)

Figure S2 Blood cell counts in DeepSAGE data captured by the eigenvector coefficients on principal components PC7 (left) and PC11 (right). Experimentally determined blood cell counts at the time of RNA isolation were available for 36/94 samples. Blood cell counts are expressed as (number of cells)×10^9^/L. Pearson correlation coefficients and corresponding p-values are shown in the plot.
(EPS)

Figure S3 Replication of Affymetrix eQTLs in DeepSAGE dataset and DeepSAGE eQTLs in Affymetrix data. The numbers of unique *cis-*regulated genes is given after each filtering step.
(EPS)

Table S1 List of detected eQTLs in tag-wise eQTL mapping.
(XLS)

Table S2 Trait-associated SNPs affecting the expression of DeepSAGE tags of 94 peripheral blood samples.
(XLS)

Table S3 List of candidate genes with alternative polyadenylation event detected using a permissive strategy.
(XLS)

Table S4 Replications between RNA-seq and DeepSAGE eQTLs.
(XLS)

Table S5 Replication of RNA-seq eQTLs in microarray-based datasets.
(XLS)

Table S6 List of detected eQTLs in the meta-analysis.
(XLS)

Table S7 Primer sequences for qPCR validation.
(XLS)

Text S1 Additional details on principal component analysis of DeepSAGE expression data.
(DOC)
