# Correction: DeepSAGE Reveals Genetic Variants Associated with Alternative Polyadenylation and Expression of Coding and Non-coding Transcripts

**DOI:** 10.1371/annotation/296056cb-f80c-4b04-985b-180f6d3cc4ae

**Published:** 2013-09-11

**Authors:** Daria V. Zhernakova, Eleonora de Klerk, Harm-Jan Westra, Anastasios Mastrokolias, Shoaib Amini, Yavuz Ariyurek, Rick Jansen, Brenda W. Penninx, Jouke J. Hottenga, Gonneke Willemsen, Eco J. de Geus, Dorret I. Boomsma, Jan H. Veldink, Leonard H. van den Berg, Cisca Wijmenga, Johan T. den Dunnen, Gert-Jan B. van Ommen, Peter A. C. 't Hoen, Lude Franke

In the Funding section, the number of the grant from the European Community's Seventh Framework Programme was missing. The sentence should read “This was partly supported by the European Community's Seventh Framework Programme (FP7/2007–2013) ENGAGE (contract no. HEALTH-F4-2007-201413)"

