# Supplementary material for: DeepSAGE Reveals Genetic Variants Associated with Alternative Polyadenylation and Expression of Coding and Non-coding Transcripts
Source: PLoS Genet. 2013 Jun 20;9(6):e1003594. doi: 10.1371/journal.pgen.1003594 (PMC3688553; doi:10.1371/journal.pgen.1003594)
Supplement: Text S1 — Additional details on principal component analysis of DeepSAGE expression data. (DOC) [file pgen.1003594.s011.doc]

**Additional details on principal component analysis of DeepSAGE expression data**

To increase the statistical power of eQTL detection, we used principal component analysis (PCA) to correct for technical and biological confounders. We determined that using 15 PCs as covariates yielded the highest number of significant *cis*-eQTLs, reflecting an almost two-fold increase.

Although correction for the first principal components substantially increased the number of detectable *cis*-eQTLs, it remains somewhat elusive why this correction procedure is so effective. We therefore investigated which phenomena these components represent and investigated the correlation with various sample characteristics. The first principal component was highly significantly correlated with the percentage of GC in the reads of a sample (r^2^ = 0.76) (Figure S1). GC content is one of the most important sources of bias in RNA-seq data and strongly affects gene expression measurements [1,2]. Although various dedicated strategies have been proposed to overcome this bias (for a review see [3]) and more sophisticated algorithms to correct for technical and biological confounders exist such as PEER and PANAMA [4–6], this straightforward PCA-based method also efficiently corrects for GC content differences across samples.

Principal components seven and eleven correlated significantly with various blood cell count parameters, indicating that these PCs reflect differences in cell type compositions between samples (Figure S2). To further substantiate this latter point, we associated the top 100 genes that had the most extreme (highest and lowest) factor loadings on PC7 and PC11 with cell types reported in the literature, using the Anni software for text concept association [7] and observed that:

- Genes with highly positive factor loadings on PC7 are strongly associated with (and therefore likely expressed in) lymphocytes. This is in agreement with the positive correlation of PC7 with lymphocyte counts (Figure S2). Genes with the most negative factor loadings on PC7 are strongly associated with macrophages and neutrophils. This is in agreement with the negative correlation of PC7 with neutrophil counts (Figure S2.

- Genes with highly positive factor loadings on PC11 are strongly associated with different types of leukocytes, while genes with the most negative factor loadings on PC11 are strongly associated with erythrocytes.

**References:**

1. Pickrell JK, Marioni JC, Pai AA, Degner JF, Engelhardt BE, et al. (2010) Understanding mechanisms underlying human gene expression variation with RNA sequencing. Nature 464: 768–772.

2. Dohm JC, Lottaz C, Borodina T, Himmelbauer H (2008) Substantial biases in ultra-short read data sets from high-throughput DNA sequencing. Nucleic Acids Res 36: e105.

3. Risso D, Schwartz K, Sherlock G, Dudoit S (2011) GC-Content Normalization for RNA-Seq Data. BMC Bioinformatics 12: 480.

4. Fusi N, Stegle O, Lawrence ND (2012) Joint modelling of confounding factors and prominent genetic regulators provides increased accuracy in genetical genomics studies. PLoS Comput Biol 8: e1002330.

5. Parts L, Stegle O, Winn J, Durbin R (2011) Joint genetic analysis of gene expression data with inferred cellular phenotypes. PLoS Genet 7: e1001276.

6. Stegle O, Parts L, Piipari M, Winn J, Durbin R (2012) Using probabilistic estimation of expression residuals (PEER) to obtain increased power and interpretability of gene expression analyses. Nat Protoc 7: 500–507.

7. Jelier R, Schuemie MJ, Veldhoven A, Dorssers LCJ, Jenster G, et al. (2008) Anni 2.0: a multipurpose text-mining tool for the life sciences. Genome Biol 9: R96.
